# Safety and Efficacy of Ranolazine for the Treatment of Chronic Angina Pectoris

**DOI:** 10.4137/CMT.S7824

**Published:** 2013-01-15

**Authors:** Mohammed Aldakkak, David F. Stowe, Amadou K.S. Camara

**Affiliations:** 1 Department of Anesthesiology, The Medical College of wisconsin, Milwaukee, WI, USA; 2 Department of physiology, The Medical College of Wisconsin, Milwaukee, WI, USA; 3 Cardiovascular research Center, The Medical College of Wisconsin, Milwaukee, WI, USA; 4 Department of Anesthesiology, Zablocki VA Medical Center research Service, Milwaukee, WI, USA; 5 Department of Biomedical Engineering, Marquette University, Milwaukee, WI, USA

**Keywords:** ranolazine, chronic angina, late Na^+^ current, mitochondria

## Abstract

Coronary heart disease is a global malady and it is the leading cause of death in the United States. Chronic stable angina is the most common manifestation of coronary heart disease and it results from the imbalance between myocardial oxygen supply and demand due to reduction in coronary blood flow. Therefore, in addition to lifestyle changes, commonly used pharmaceutical treatments for angina (nitrates, β-blockers, Ca^2+^ channel blockers) are aimed at increasing blood flow or decreasing O_2_ demand. However, patients may continue to experience symptoms of angina. Ranolazine is a relatively new drug with anti-anginal and anti-arrhythmic effects. Its anti-anginal mechanism is not clearly understood but the general consensus is that ranolazine brings about its anti-anginal effects by inhibiting the late Na^+^ current and the subsequent intracellular Ca^2+^ accumulation. Recent studies suggest other effects of ranolazine that may explain its anti-anginal and anti-arrhythmic effects. Nonetheless, clinical trials have proven the efficacy of ranolazine in treating chronic angina. It has been shown to be ineffective, however, in treating acute coronary syndrome patients. Ranolazine is a safe drug with minimal side effects. It is metabolized mainly in the liver and cleared by the kidney. Therefore, caution must be taken in patients with impaired hepatic or renal function. Due to its efficacy and safety, ranolazine was approved for the treatment of chronic angina by the Food and Drug Administration (FDA) in 2006.

## Introduction

Cardiovascular diseases are the number one cause of death globally. According to the World Health Organization, an estimated 17.3 million people died from cardiovascular diseases in 2008, representing 30% of all global deaths. Of these deaths, an estimated 7.3 million were due to coronary heart disease. Coronary heart disease, along with stroke, is projected to remain the single leading cause of death in the future. Chronic stable angina is the most prevalent manifestation of coronary artery disease (CAD) with an incidence of 320,000 cases in males and 180,000 cases in females every year in the US.^1^ Stable angina is a clinical syndrome characterized by discomfort in the chest, jaw, shoulder, back, or arms, typically elicited by exertion or emotional stress and relieved by rest or nitroglycerin,^2^ and it is caused by an imbalance between myocardial oxygen supply and myocardial oxygen consumption. Management of chronic stable angina includes lifestyle changes (eg, eating a heart-healthy diet, lowering cholesterol, getting regular exercise, cessation of smoking, and controlling diabetes and high blood pressure), pharmacological treatment (β-blockers, Ca^2+^ channel blockers, nitrates, and antiplatelet medications), and revascularization procedures when indicated (percutaneous coronary intervention (PCI), coronary artery bypass grafting).^2^ Despite the effectiveness of the current management strategies, episodes of angina may still persist or even worsen^3,4^ and many patients cannot tolerate a combination of anti-anginal drugs due to their many side effects. Therefore, a new treatment that provides an alternative option for patients who continue to suffer from symptoms of angina despite using other anti-anginal drugs would be helpful. Ranolazine is a relatively new drug for the treatment of chronic angina that was approved by the Food and Drug Administration (FDA) and has been in use in the US since 2006.

## Metabolism and Pharmacokinetic Profile

Ranolazine is a racemic mixture, chemically described as 1-piperazineacetamide, N-(2,6-dimethylphenyl)-4-[2-hydroxy-3-(2-methoxyphenoxy)propyl], with the empirical formula of C_24_H_33_N_3_O_4_.^5^ It contains two enantiomeric forms (S-ranolazine and R-ranolazine).^6^ Ranolazine was initially manufactured in an immediate-release formulation but it was discontinued due to the short elimination half life.^7^ Ranolazine is currently manufactured by Gilead Sciences in an extended (sustained)-release tablets of 500 mg and 1000 mg for clinical use and it is sold under the trade name Ranexa^®^. It is extensively absorbed after oral administration and it reaches peak plasma concentrations within 2–5 hours^5,7^ after a single dose where it binds mainly to α-1 acid glycoprotein and weakly to albumin. With a dose of 500–1000 mg given twice daily, the peak to trough difference is only 1.6 fold. Steady state is usually reached within 3 days of multiple dosing. The half-life of ranolazine at steady state after oral administration is nearly 7 hours. Less than 5% of ranolazine is excreted unchanged by the kidney while the remainder is extensively and rapidly metabolized mainly by CYP3A4 enzymes in the liver and, to a lesser extent, by CYP2D6. Following a single oral dose of ranolazine, total elimination is 75% in urine and 25% in feces. These pharmacokinetics are not affected by age, gender, or food.^5^

## Mechanism of Action

Although the precise mechanisms for the anti-anginal effects of ranolazine are not well understood, several pharmacological activities of ranolazine have been described. Of these, two distinct mechanisms appear to primarily promote its cardiac protection against ischemic injury. These include its effect as a metabolic modulator and as a late Na^+^ current inhibitor. Therefore, in this section we will summarize the findings of the many studies in which attempts were made to elucidate the mechanism of action of ranolazine. Table 1 summarizes some of the mechanisms of ranolazine that could be responsible for its anti-anginal effects.

### Ranolazine as a metabolic modulator

In general, drugs such as β-blockers, Ca^2+^ channel blockers, and long acting nitrates exert their anti-anginal effects by causing hemodynamic changes such as drop in blood pressure, heart rate, and/or contractility which decreases cardiac work.^8^ On the other hand, ranolazine as many trials showed, does not have any clinically significant effect on these hemodynamic parameters.^9–14^ Initially it was reported that ranolazine was effective as an anti-anginal drug by acting as a metabolic modulator.^15^ For this mechanism, ranolazine was proposed to stimulate glucose oxidation at the expense of fatty acid oxidation. Under non-ischemic conditions, myocytes ATP requirement is met by both glucose and fatty acid oxidation with the majority (about 90%) coming from fatty acid oxidation.^16^ Fatty acid oxidation is more efficient, energy-wise, than glucose oxidation in ATP production. However as the supply of O_2_ dwindles during ischemia, glucose becomes the preferred substrate for ATP production because it is more efficient oxygen-wise (for more details on this topic refer to the review by Cheng et al^16^). Therefore, by switching substrate utilization to glucose oxidation, ranolazine may provide a non-hemodynamic mechanism for preserving cardiac myocytes during ischemia and reperfusion injury. It is not clear how ranolazine promotes glucose oxidation, but in perfused guinea pig hearts subjected to low-flow ischemia it was shown that ranolazine increased the amount of active dephosphorylated pyruvate dehydrogenase^15^ which is thought to play a key role in determining the rate of carbohydrate utilization.^17,18^

### Ranolazine as a late Na^+^ current inhibitor

The effect of ranolazine as a modulator of metabolism occurs at concentrations greater than 10 μM. However, ranolazine exerts its effects as an anti-anginal drug at lower concentrations, ie, 2–6 μM.^19^ This implies that a mechanism other than promoting glucose oxidation is probably responsible for the anti-anginal characteristics of ranolazine. Indeed, in a comprehensive study^19^ it was shown that ranolazine produces several ion channel effects. Of particular interest was the inhibitory effect of ranolazine on the late Na^+^ current (*I*Na_L_) with an IC_50_ = 5.9 μM.^19^ This inhibitory effect was also observed in other studies in which the *I*Na_L_ was pharmacologically induced by the *Anemonia sulcata* toxin (ATX)-II.^20,21^ A later study showed that ranolazine blocked both the peak Na^+^ current (*I*Na) and the *I*Na_L_ with a nine-fold selectivity for *I*Na_L_ vs. *I*Na,^22^ and that this blockade occurred at the same binding site where other local anesthetics, such as lidocaine, interacts with Na^+^ channels. Although the inhibitory effect of ranolazine on Na^+^ current and especially on the *I*Na_L_ may suggest an anti-arrhythmic effect, it could also explain its mechanism as an anti-anginal drug on the basis of preventing Na^+^ overload and the subsequent increase in intracellular Ca^2+^.

Cytosolic Ca^2+^ overload is a key event in the injury sustained during ischemia. It occurs due to activation of the Na^+^/Ca^2+^ exchanger in the reverse mode as a result of the increase in cytosolic Na^+^ which results from impaired Na^+^/K^+^ ATPase pump activity, coupled with an increased pH gradient between the intracellular and extracellular spaces and concomitant activation of the Na^+^/H^+^ exchanger.^23^ However, increased cytosolic Na^+^ may also arise in part from activation of the *I*Na_L_ by toxic ischemic metabolites^24,25^ and by reactive oxygen species that are generated during reperfusion.^26^ Thus ranolazine, an inhibitor of *I*Na_L_, could reduce cytosolic Na^+^ during ischemia and subsequently reduce cytosolic Ca^2+^. Indeed, Song et al^27^ reported that ranolazine attenuated H_2_O_2_-induced intracellular Na^+^ and Ca^2+^ overload in rabbit isolated ventricular myocytes. We also showed that ranolazine reduced cytosolic Ca^2+^ in intact beating guinea pig hearts undergoing global no flow ischemia, and that this led to a decrease in mitochondrial Ca^2+^ overload and reactive oxygen species generation during ischemia.^28^ These effects of ranolazine on mitochondria may underlie, in part, its cardio-protective effects against ischemia. What remains unclear is the exact mechanism by which ranolazine inhibits *I*Na_L_.

Recent studies have focused on the pore-forming subunit of the cardiac Na^+^ channel, Na*v*1.5, which is the main ion channel that conducts Na^+^ in cardiac cells. One interesting study showed mechano-sensitive properties for *I*Na in isolated mouse cardiomyocytes.^29^ This implies that the function of Na*v*1.5, among many other described regulatory mechanisms, is also modulated by the mechanical stretch of the membrane in which it is embedded. However, and more importantly, the novelty of the study came from the finding that ranolazine inhibited the mechano-sensitivity of Na*v*1.5 in a dose dependent manner, and that this inhibition did not require the established binding site. This study also suggested that it is only the neutral form of ranolazine that is capable of inhibiting the mechano-sensitivity of Na*v*1.5, thus leading the authors to speculate that ranolazine obtains access to the channel by partitioning within the lipid bilayer of the cell membrane. Ranolazine inhibited the mechano-sensitivity of Na*v*1.5 with an IC_50_ = 54 μM which is about 10-fold higher than the therapeutic concentration. Nonetheless, the authors suggest two reasons not to dismiss these findings. First, the results were obtained in an experimental model that does not mimic exactly the true mechanical environment of the channels in situ where other mechano-relevant elements likely mediate the mechano-sensitivity of the Na*v*1.5. Second, ranolazine inhibits the mechano-sensitivity of Na*v*1.5 by partitioning into the bilayer hydrophobic core^29^ in its neutral (effective) form. However, this partitioning increases with increasing temperatures and since the experiments were conducted at room temperature, one can expect that the effective membrane concentration of ranolazine will increase at the higher physiological temperatures of the clinical setting.

### Ranolazine and mitochondrial complex I

As mentioned earlier, the effect of ranolazine as a metabolic modulator is probably related to increasing the amount of the active dephosphorylated pyruvate dehydrogenase. It is worth noting, however, that the pyruvate dehydrogenase complex is exclusively intramitochondrial in mammalian tissues, which may suggest that ranolazine has another site of action that is intra-mitochondrial.^8^ Interestingly, Wyatt et al^30^ showed that ranolazine inhibited mitochondrial respiration of NADH-linked substrates and that this was due to inhibition of the mitochondrial electron transport chain at the level of complex I. Furthermore, the inhibition by ranolazine resembled that of rotenone and amobarbital which are specific complex I blockers. The significance of this is that inhibiting complex I with amobarbital was protective against ischemic injury as we and others have shown.^31,32^ We postulated that inhibiting complex I during ischemia decreases electron transfer from complex I to complex III, thereby reduces electron leak, and superoxide (O_2_^•−^) generation at complex III during late ischemia. So, if ranolazine inhibits complex I in a manner similar to that of rotenone and amobarbital, then it could very well promote its anti-anginal effect in part by a mitochondrial mediated mechanism, which involves the reduction in reactive oxygen species generation during ischemia and subsequently on reperfusion.

Indeed, ranolazine was shown to reduce reactive oxygen species generation during ischemia.^28^ Furthermore, in a recent study Gadicherla et al showed that in hearts exposed to global ischemia, the addition of ranolazine on reperfusion improved mitochondrial complex I activity, restored electron transfer through Fe-S clusters of complex I, and preserved supercomplex assembly and cardiolipin integrity.^33^ However, there was no evidence that these observations were due to direct effects of ranolazine on complex I or even on mitochondria. Therefore, it was concluded that ranolazine indiscriminately reduced oxidative damage to complex I and its supportive structures and thus maintained optimal electron flow through complex I and reduced the tendency for electron leak and the subsequent generation of O_2_^•−^. It is important to mention that in the study by Wyatt et al^30^ ranolazine was shown to be a weak inhibitor of complex I when compared with rotenone or amobarbital, especially in energetically coupled mitochondria. However, in uncoupled or broken mitochondria (sub-mitochondrial particles) ranolazine exerted a more potent inhibitory effect on complex I. The authors suggested that this inhibitory effect by ranolazine occurred because of the lower membrane potential in uncoupled mitochondria coupled with a more acidic environment which favors greater protonation and uptake of ranolazine, or that it is only the charged form of ranolazine that is inhibitory. Nonetheless, regardless of the conditions of energisation of the mitochondrial inner membrane, the IC_50_ for inhibition of mitochondrial complex I by ranolazine was much higher than its therapeutic concentrations.

### Ranolazine modulates myofilaments Ca^2+^ sensitivity

The general consensus so far is that ranolazine acts as an anti-anginal and anti-ischemic drug primarily by inhibiting *I*Na_L_. In doing so, ranolazine prevents cytosolic Na^+^ overload and the subsequent excess in cytosolic Ca^2+^. The increase in cytosolic Ca^2+^ plays a key role in ischemic injury by causing mitochondrial Ca^2+^ overload, which then triggers a cascade of events that eventually leads to apoptosis/necrosis and cell death.^34,35^ Increase in cytosolic Ca^2+^ may induce damage by non-mitochondrial mechanisms such as by activating Ca^2+^-dependent phospholipases and proteases with the subsequent loss of structure and function of the myocyte.^36^ Ca^2+^ also may induce diastolic dysfunction which leads to a sustained contracture during diastole, an increase in myocardial O_2_ consumption and compression of intramural small vessels, thus reducing nutritive blood flow to the ischemic territory.^37^ Interestingly ranolazine was found to attenuate the diastolic dysfunction in several animal studies^38–43^ and in human studies.^44,45^ While ranolazine may reduce diastolic dysfunction by inhibiting *I*Na_L_ and the subsequent increase in cytosolic Ca^2+^, a very recent study may shed some light on a different mechanism for ranolazine.

In this study,^46^ the authors used a hypertensive mouse model (deoxycorticosterone acetate [DOCA]-salt) of diastolic dysfunction. It is important to mention that, in this study, no *I*Na_L_ was noted, nor were there changes in Ca^2+^ cycling to indicate significant alterations in Ca^2+^ handling in this form of diastolic dysfunction. Yet ranolazine was effective in abating this form of diastolic dysfunction. Myofilaments isolated from mice with diastolic dysfunction exhibited increased maximal tension and sensitivity to Ca^2+^ as well as a slowing of cross-bridge exit kinetics compared with sham mice, and these changes were normalized with ranolazine treatment. This suggested that ranolazine improved diastolic function at the cardiomyocyte level through the modulation of myofilament cross-bridge kinetics and sensitivity to Ca^2+^. Additionally, ranolazine, acting directly on myofilaments, improved diastolic dysfunction in the DOCA-salt sensitive mice with the same concentrations used clinically with no significant effects on hemodynamic parameters. The results obtained from the DOCA-salt sensitive mice may not be transferable to humans. Nonetheless, the finding of this study may hint to a new possible mechanism of ranolazine as an anti-anginal drug which deserves further exploration.

## Clinical Studies

Several clinical trials have studied the effects of ranolazine in patients with coronary heart disease. Of these, three major trials focused on the role of ranolazine in treating chronic stable angina and one trial study focused on the role of ranolazine in treating acute coronary syndrome. A recent clinical trial focused on the role of ranolazine in PCI, and one more study assessed the cost of using ranolazine. There are no further studies assessing the effects of ranolazine on cardiovascular morbidity in patients with chronic angina. These studies are summarized below.

### MARISA trial

**Figure 1:**
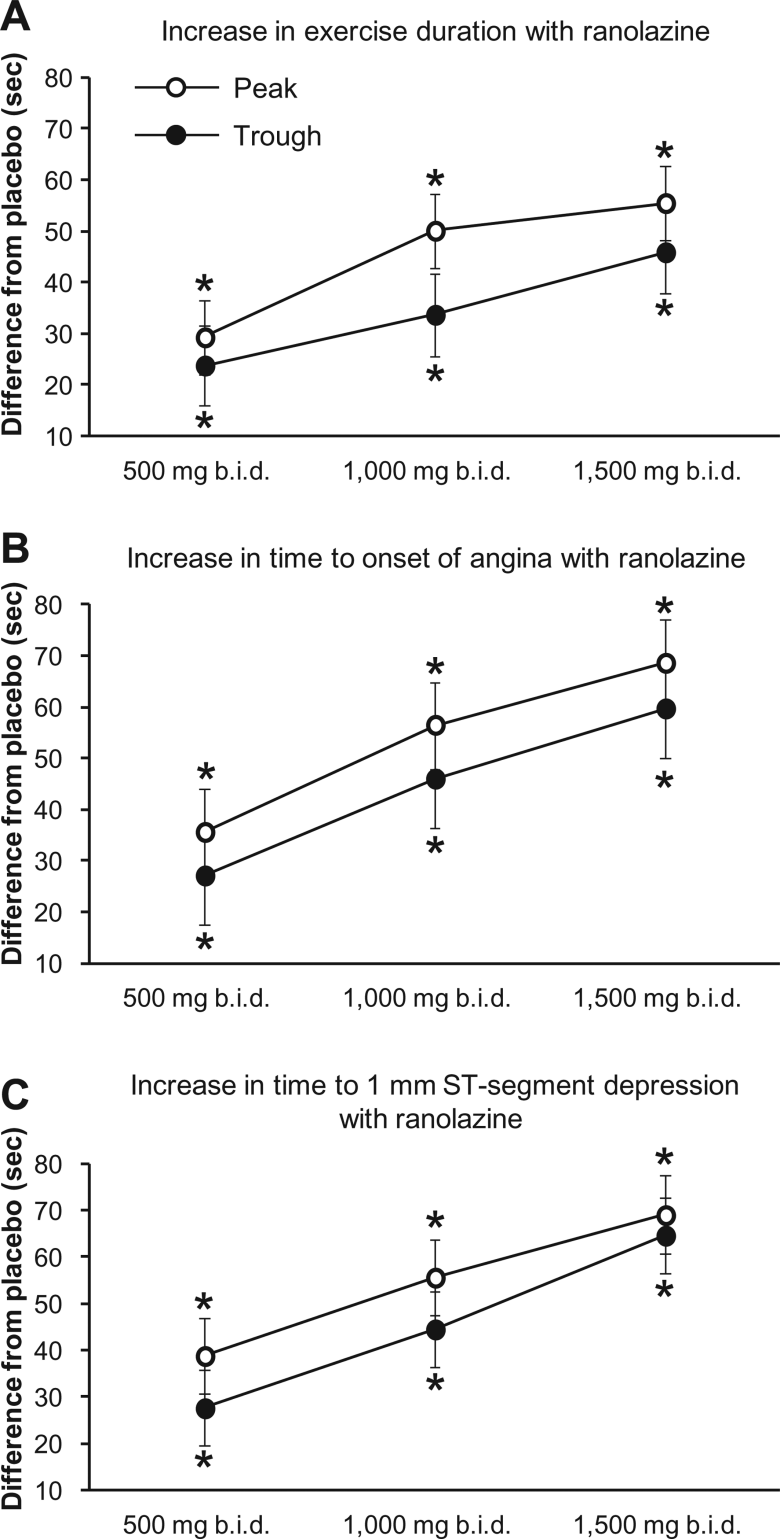
Summary of the effects of three doses of ranolazine on exercise treadmill test parameters. **Notes:** values shown represent difference from placebo in seconds. Data are mean ± standard error. *P < 0.05 in treatment with ranolazine vs. placebo. b.i.d. = twice daily.

Before the MARISA (Monotherapy Assessment of Ranolazine In Stable Angina) trial, clinical studies focused on the immediate release formulation of ranolazine in combination with other anti-anginal drugs. The MARISA trial was the first study to focus on the sustained release formulation of ranolazine as a sole treatment for angina. The purpose of the study was to compare, in a dose dependent manner, the effects of the sustained release formulation of ranolazine on treadmill exercise performance and to provide long-term survival data in patients who completed the trial.^10^ In this study, 191 patients with documented CAD and treated with anti-anginal drugs were enrolled. These patients had to discontinue their anti-anginal treatments during the study except for sublingual nitroglycerin as needed. Randomized patients received a double-blind treatment with the sustained release formulation of ranolazine at doses of 500, 1000, and 1500 mg or a placebo, each administered twice daily for one week, according to a four-period, balanced Latin Square crossover design. At the end of each treatment period, exercise treadmill tests were performed at 4 h (peak) and later at 12 h (trough) after dosing. Compared to placebo, treatment with ranolazine significantly improved total exercise duration, time to angina, and time to 1 mm ST-segment depression at both peak and trough time points in a dose dependent manner (Fig. 1). Increase in ranolazine plasma concentrations was associated with an increase in mean exercise durations, but the increase in exercise duration appeared to plateau at the higher ranolazine concentrations. Further analysis showed that gender, age, diabetes, and prior history of heart failure did not significantly modify the treatment effects of ranolazine. Some patients from the MARISA trial were still receiving ranolazine at one year and two years after their first doses. Their one-year and two-year survival rates on ranolazine were 96.3% and 93.6%, respectively. The MARISA study showed also that ranolazine was a safe drug with side effects usually occurring at higher dosage. Common side effects included constipation, nausea, dizziness, and asthenia. Ranolazine also prolonged the corrected QT (QT_c_) interval in a dose dependent manner. In summary, the MARISA trial showed that ranolazine is an effective and safe drug when used alone as an anti-anginal treatment.

### CARISA trial

**Figure 2:**
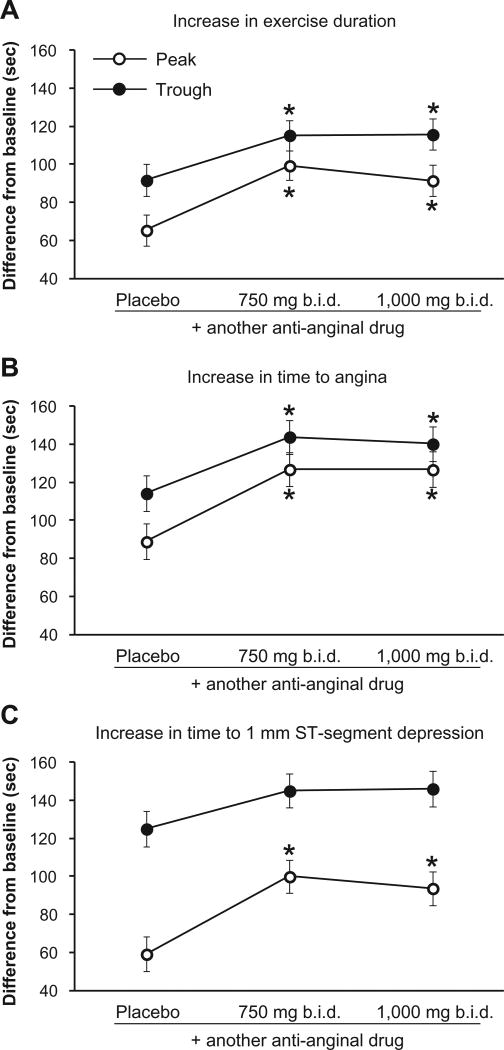
Summary of the effects of two doses of ranolazine on exercise treadmill test parameters. **Notes:** values shown represent difference from baseline values in seconds. placebo indicates treatment only with another anti-anginal drug (atenolol 50 mg, amlodipine 5 mg, diltiazem 180 mg). Data are mean ± standard error. *P < 0.05 in treatment with ranolazine vs. placebo. b.i.d. = twice daily.

**Figure 3:**
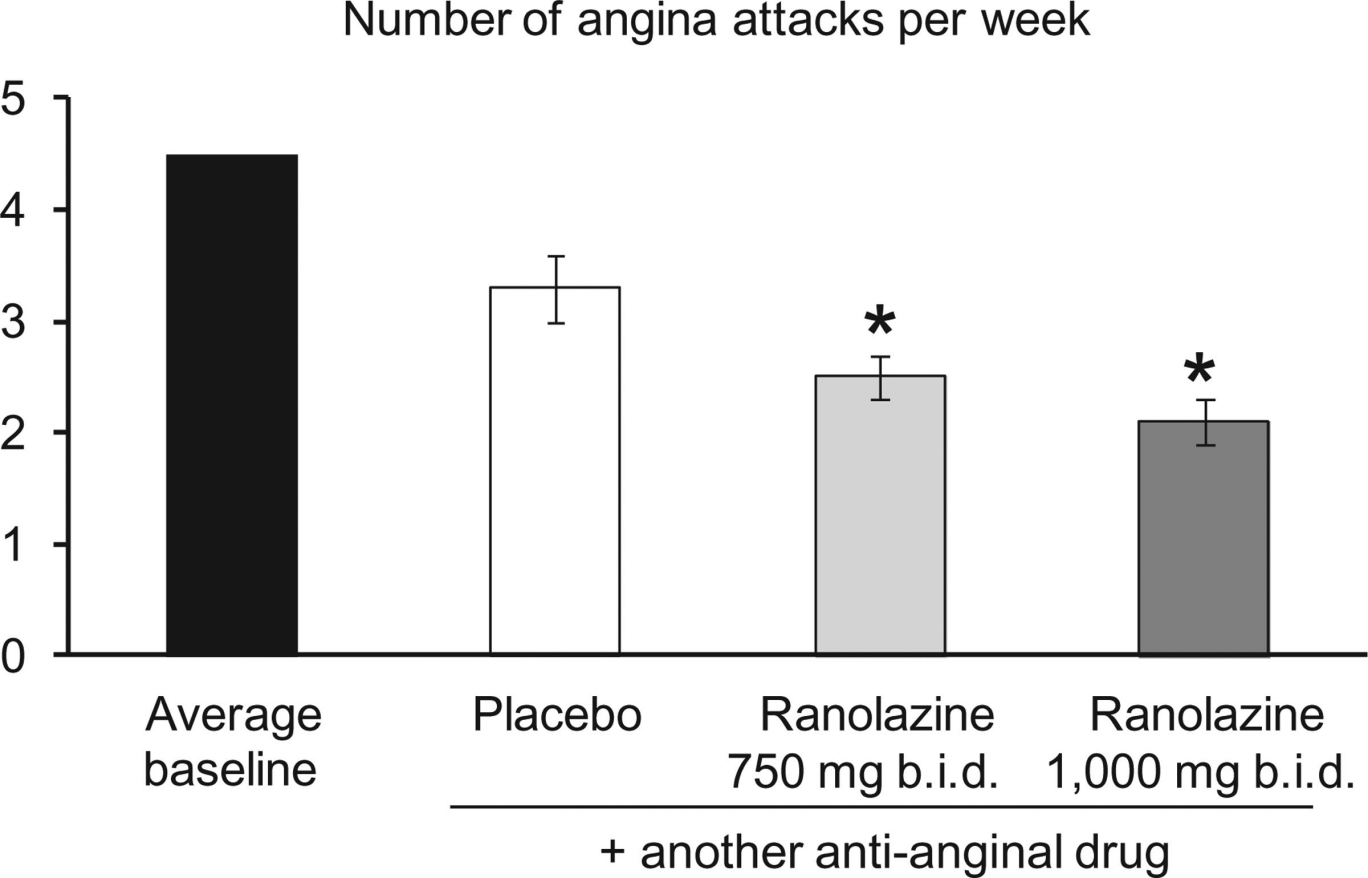
Summary of the effects of two doses of ranolazine on number of angina attacks per week. **Notes:** Placebo indicates treatment only with another anti-anginal drug (atenolol 50 mg, amlodipine 5 mg, diltiazem 180 mg). Data are mean ± standard error. *P < 0.05 in treatment with ranolazine vs. placebo. b.i.d. = twice daily.

The purpose of the CARISA (Combination Assessment of Ranolazine In Stable Angina) trial was to assess the anti-anginal and anti-ischemic effects of ranolazine in symptomatic chronic angina patients with severe CAD when combined with standard doses of a typical anti-anginal drug, ie, atenolol, amlodipine, or diltiazem.^9^ The efficacy end points included treadmill exercise duration, time to angina, time to 1 mm ST-segment depression at peak and trough, and the number of angina attacks and sublingual nitroglycerin uses reported by the patients. In this study, 823 patients were assigned randomly to receive placebo, 750 mg ranolazine, or 1000 mg ranolazine twice daily for 12 weeks in addition to receiving another typical anti-anginal drug. The major finding was that ranolazine at the two doses significantly increased exercise duration at both the trough and the peak concentrations (Fig. 2). More importantly, treatment with the other anti-anginal drugs did not significantly modify the response to ranolazine. Ranolazine decreased the number of angina attacks and subsequently it decreased nitroglycerin consumption (Fig. 3). Some patients from the CARISA trial were still receiving ranolazine at one year and two years after their first doses. Their one-year and two-year survival rates on ranolazine were 98.4% and 95.9%, respectively. As in the MARISA trial, common side effects included constipation, nausea, dizziness, and asthenia with very few patients using 1000 mg ranolazine reporting episodes of syncope. Ranolazine also caused small increases in QT_c_ interval. In summary, the CARISA trial showed that ranolazine can provide an additional anti-anginal effect in patients treated with the classical anti-anginal drugs.

### ERICA trial

**Figure 4:**
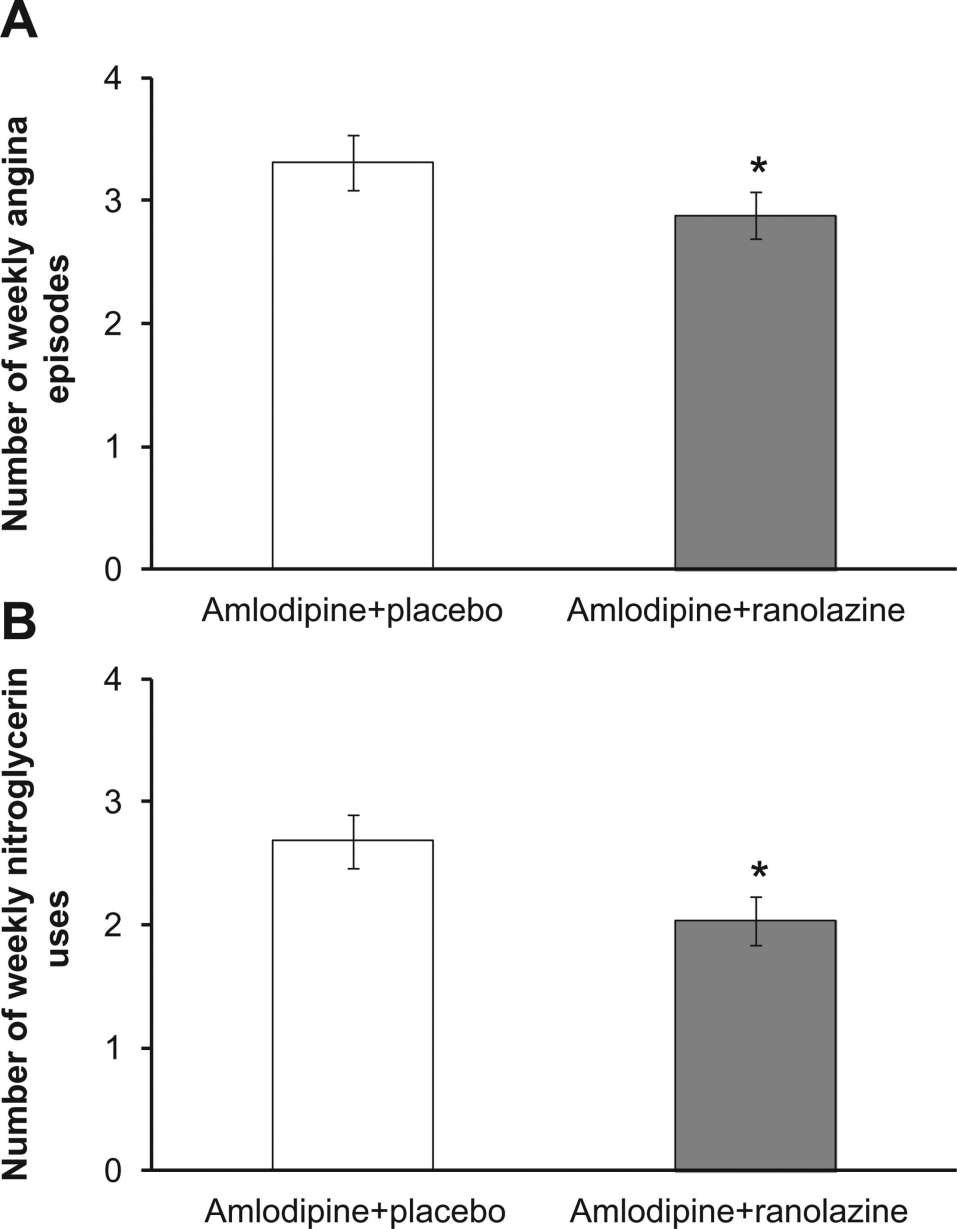
Summary of the effects of ranolazine when added to a maximum dose of amlodipine (10 mg) on the number of angina episodes and nitroglycerin uses per week. **Notes:** Data are mean ± standard error. *P < 0.05 in treatment with ranolazine vs. placebo.

**Figure 5:**
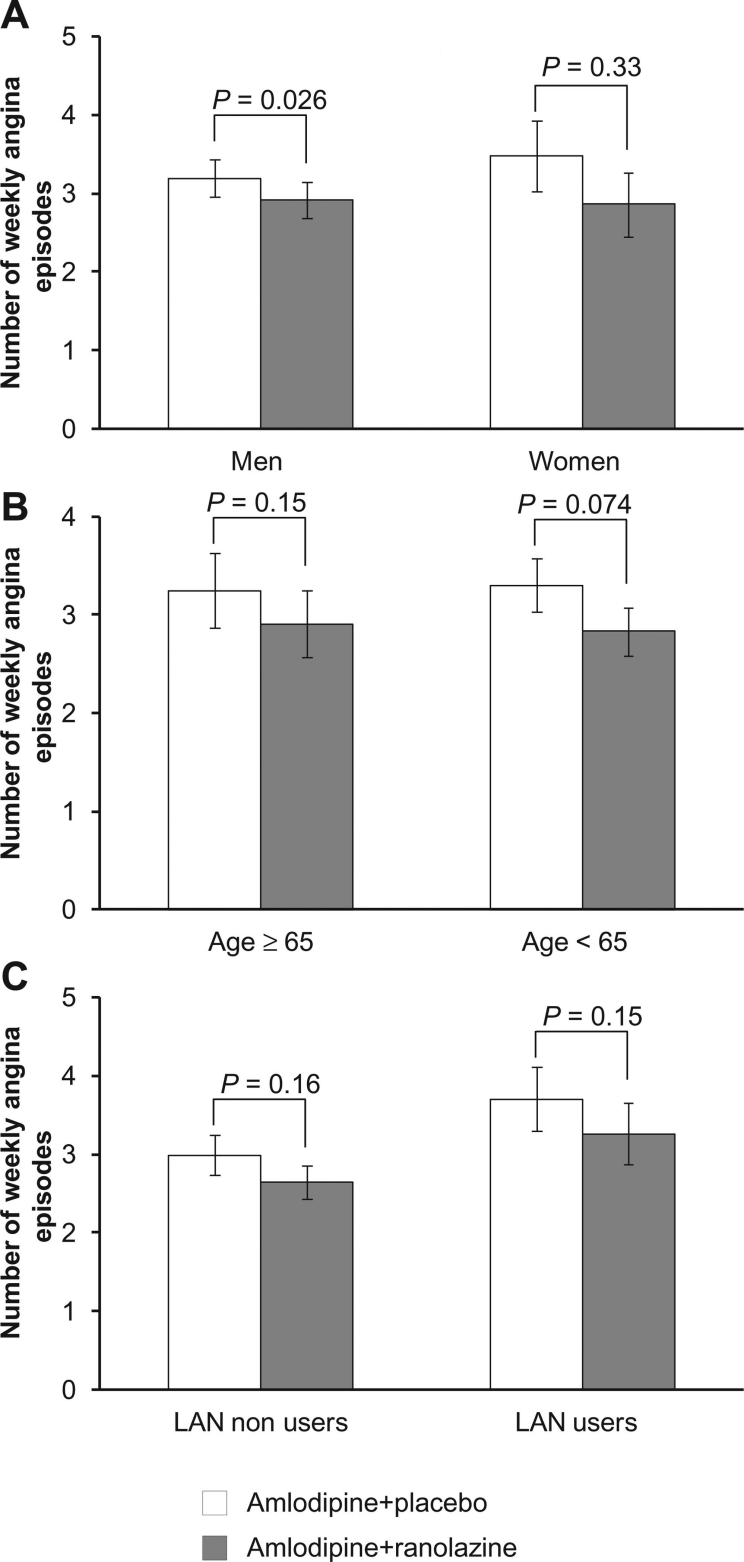
Summary of the effects of ranolazine when added to a maximum dose of amlodipine (10 mg) on the number of angina episodes per week in three subgroups. **Note:** Data are mean ± standard error. **Abbreviation:** LAN, long acting nitrate.

Prior to the ERICA (Efficacy of Ranolazine In Chronic Angina) trial, ranolazine was shown to be effective as an anti-anginal treatment when used alone or in combination with other anti-anginal drugs at sub-maximal dosage, but its efficacy when combined with a maximum recommended dosage of another conventional anti-anginal treatment had not been investigated. Therefore, the goal of the ERICA trial was to determine if ranolazine could reduce angina in patients with persistent angina despite treatment with the maximal recommended daily dosage of amlodipine.^14^ The efficacy of ranolazine was assessed by the weekly average frequency of angina episodes, the average weekly nitroglycerin consumption rate, and the change from baseline of the 5 dimensions of the Seattle Angina Questionnaire (SAQ). Safety was assessed by evaluating reported adverse effects, hemodynamics, laboratory measures, and ECG. In this study, 565 patients with ≥3 episodes of angina per week were receiving a maximum dosage of amlodipine at 10 mg/day. In addition to amlodipine, patients were randomized to also receive either 1000 mg ranolazine or placebo twice daily for 6 weeks. Adding ranolazine reduced the weekly rate of angina episodes (Fig. 4A), and it reduced the average weekly rate of nitroglycerin consumption (Fig. 4B), but it improved only the angina frequency dimension of the SAQ. The treatment effect of ranolazine in different subgroups was numerically similar to that in the population as a whole (Fig. 5), however the study was not powered to test the treatment effects within subgroups. Ranolazine did not induce any significant changes in heart rate or blood pressure when compared to placebo. The adverse effects occurred in 35.3% of amlodipine+placebo and 39.9% of amlodipine+ranolazine-treated patients with women and patients older than 65 reporting more adverse effects. In summary, the ERICA trial demonstrated that ranolazine provided additional anti-anginal benefit in patients who remained symptomatic despite being treated with the maximal dosage of the Ca^2+^ channel blocker amlodipine.

### MERLIN-TIMI 36 trial

The goal of the MERLIN (Metabolic Efficiency with Ranolazine for Less Ischemia in Non-ST-Elevation Acute Coronary Syndrome)-TIMI 36 trial was to evaluate the efficacy and safety of ranolazine in the short and long term in patients with acute coronary syndromes who were also receiving standard therapy.^47^ The primary efficacy end point was a composite of cardiovascular death, myocardial infarction, or recurrent ischemia through the end of the study. The major safety end points were assessed as death from any cause and the incidence of symptomatic documented arrhythmia. In this study, 6560 patients who were receiving standard treatment for non-ST elevation acute coronary syndrome were also randomized to additionally receive ranolazine or placebo. Ranolazine 200 mg (or matching placebo) was given first intravenously within 1 h followed by slower intravenous infusion over the next 12–96 h. Then, ranolazine extended-release (or matching placebo) tablet was given orally at a dose of 1000 mg twice daily for the remainder of the study (up to 24 months with a median of 348 days). Ranolazine did not reduce the rate of cardiovascular death, myocardial infarction, or recurrence of ischemia (ranolazine 21.8% vs. placebo 23.5%, *P* = 0.11), all considered as the primary endpoints of the study. However, the effect of long term treatment with ranolazine on angina was evident as it reduced worsening angina (ranolazine 4.2% vs. placebo 5.9%, *P* = 0.02). Moreover, an increase in or addition of an anti-anginal drug was less frequent (ranolazine 10.6% vs. placebo 13%, *P* = 0.003). There was no significant heterogeneity in the effect of ranolazine on the primary endpoints across the major subgroups examined. Death from any cause did not differ among patients treated with ranolazine compared with patients treated with placebo. The incidence of symptomatic documented arrhythmias throughout the duration of the study was similar in patients treated with ranolazine compared with placebo. However, the frequency of clinically significant arrhythmias observed during Holter monitoring during the first 7 days was lower in the ranolazine group vs. in the placebo group. Adverse events (dizziness, nausea, constipation, syncope) were more frequent with ranolazine vs. placebo. In summary, this study showed no significant benefit of ranolazine compared with placebo with respect to the primary efficacy endpoints. Also, ranolazine appeared to be safe but with no difference from placebo with respect to the safety endpoints.

### Role of ranolazine during percutaneous coronary intervention

Myocardial injury may occur in patients undergoing PCI and this may lead to higher mortality. Therefore, the goal of this study was to determine if pretreatment with ranolazine before PCI has any protective role on periprocedural myocardial damage.^48^ The primary endpoint was defined as the postprocedural increase in creatine kinase-MB (CK-MB) ≥3 times of the upper limit of normal indicating periprocedural myocardial damage. Other cardiac markers such as Troponin I and myoglobin levels were also measured. In this study, 70 patients who fulfilled the inclusion criteria and scheduled for elective PCI were included and randomly divided into two groups, 35 patients each. One group received placebo while the other received ranolazine 1000 mg twice daily for 7 days before the planned intervention. Ranolazine reduced the periprocedural myocardial infarction (ranolazine 6% vs. placebo 22%, *P* = 0.041), which is the primary endpoint. Overall, there were fewer patients in the ranolazine group compared to the placebo group that showed increases above the upper limit of normal in CK-MB (ranolazine 23% vs. placebo 40%, *P* = 0.01), troponin I (ranolazine 31% vs. placebo 48%, *P* = 0.011), and myoglobin (ranolazine 28% vs. placebo 43%, *P* = 0.033). Further, ranolazine reduced the incidence of death, myocardial infarction, or target-vessel revascularization at 30 days after the procedure (ranolazine 9% vs. placebo 28%, *P* = 0.03), but this was mainly influenced by the periprocedural events because when periprocedural infarctions were not counted there was no significant difference between the two groups (ranolazine 3% vs. placebo 6%, *P* = 1). In summary, this study showed that pretreatment with ranolazine is effective in decreasing the incidence of myocardial infarction during PCI.

### Costs and clinical outcome

Ranolazine per dose is more expensive than the other traditional anti-anginal treatments. However, the focus should be on total costs of care and not on the cost of single treatment. Although some studies analyzed the costs for treating angina, the study by Phelps et al^49^ was the first chronic angina medication-specific cost comparative study. In this study, a large managed care administrative claims database was analyzed to better understand the effect of ranolazine versus alternative anti-anginal drugs on health care use and total costs of care. Specifically, the primary dependent variables for this study were total costs of care and rate of revascularization procedures and services. A retrospective administrative claims-based analysis from a large, geographically diverse US health insurance plan with 11.2 million commercial enrollees was performed. First, patients who were identified to have angina were selected. Then of these, only patients who were prescribed a new anti-anginal drug (index event) that was not used within the previous 6 months (pre-index period) and continued using the new treatment for 6 more months (post-index period) were selected. This was to insure that only patients who had uncontrolled angina or had side effects that required their physician to alter their medication plan were enrolled. After further exclusion, 4545 patients were left for final analysis and were divided into 3 groups. Newly prescribed ranolazine, long acting nitrates, or β-blockers/Ca^2+^ channel blockers. In the 6 months pre-index period, there were no significant differences among all groups in total costs of care and in the rates of revascularization. Interestingly, even though ranolazine is more expensive per dose than the other anti-anginal drugs, the unadjusted total costs of care were lower for ranolazine than for the other groups. This is likely a result of fewer hospitalizations in patients treated with ranolazine compared to the other non-ranolazine treated groups. The ranolazine treated group also had significantly lower revascularization rates in the 6 months post-index period. Furthermore, a multivariate analysis was performed to control for differences in age, gender, and presence of co-morbid conditions that can affect total costs of care. But even then, the adjusted total costs of care and revascularization rates were significantly lower in the ranolazine treated group compared to the other non-ranolazine treated groups. Therefore, it was concluded that adding ranolazine to the therapy of patients with poorly controlled angina could lead to better outcome and lower costs compared with the other alternatives. These finding are all summarized in Table 2.

## Safety

From all the 4 major clinical trials, ranolazine, at any dose, did not induce clinically significant changes in heart rate or blood pressure during rest or exercise. Overall, the side effects event rate increased with higher doses. The most common side effects of ranolazine are dizziness, nausea, vomiting, constipation, headache, and asthenia. However, all these side effects are mild to moderate in general, occur soon after using the drug, and reverse promptly with reducing the dose or discontinuation of treatment.^50^ The increase in adverse events seen with the 1500 mg dose in MARISA is disproportionately larger than the increase in anti-anginal efficacy; thus, the 1500 mg twice-daily dose is not recommended for clinical use. Postural hypotension and syncope were observed in a small number of patients taking ≥ 1000 mg ranolazine, most likely due to concomitant usage of other medications that are vasoactive or known to increase the plasma concentration of ranolazine. However, this can be prevented by starting ranolazine at a low dose (500 mg) and slowly increasing the dose as needed. Ranolazine may cause some small changes in laboratory parameters such as an increase in eosinophil count, an increase in creatinine, a decrease in hematocrit, and a decrease in hemoglobin A_1C_ in diabetic patients. Ranolazine may prolong the QT_c_ interval in a dose dependent manner,^9,10^ therefore it is recommended to avoid ranolazine in patients with known long QT_c_ and in patients taking drugs that may prolong QT_c_.

Ranolazine is metabolized primarily in the liver by the enzyme CYP3A4, therefore caution should be taken in those who use other drugs that are known to interact with this enzyme or in patients with hepatic dysfunction as this may cause accumulation of ranolazine.^51^ Furthermore, ranolazine is cleared mainly by the kidneys and so careful dose titration is recommended in patients with mild to moderate renal impairment, whereas it is completely contraindicated in patients with severe renal impairment.^52^ Ranolazine is transported by P-glycoprotein^7^ and therefore it must be used cautiously with drugs that inhibit P-glycoprotein such as verapamil, as this may increase the absorption and subsequently the plasma concentration of ranolazine.

## Efficacy

Early on, ranolazine was manufactured in an immediate release form and several clinical trials tested its efficacy for the treatment of chronic angina.^11–13,53,54^ These trials yielded results ranging from negative effects to studies showing benefits of ranolazine as an anti-anginal treatment. This range of effects likely stems from the different dosages utilized. Eventually, the immediate release form was replaced by the more favorable extended (sustained) release form which is now commercially available for treatment. The efficacy of this formulation was evaluated in the 4 large clinical trials summarized above.^9,10,14,47^ MERLIN-TIMI 36 was the only study to assess the efficacy of the extended formulation of ranolazine for treating non-ST-elevation acute coronary syndrome and it showed no benefit for ranolazine in this population. However, the other three studies (MARISA, CARISA, ERICA) demonstrated beneficial effects of ranolazine in treating chronic angina. For example, ranolazine alone (MARISA) increased the treadmill exercise performance similar to that observed with other anti-anginal drugs.^55,56^ However, when ranolazine was combined with other anti-anginal drugs in some trials (CARISA, ERICA), the beneficial effects of ranolazine seemed initially limited, ie, it reduced angina episodes by nearly 1 per week, and it increased exercise duration by nearly 24–34 s at trough concentrations (MARISA) compared to placebo. Nonetheless, one cannot underestimate these findings because a small increase in exercise duration translates into the ability of patients to carry on more daily living activities without being symptomatic. Therefore, and based on these findings, it is safe to say that ranolazine is recommended for the therapy of chronic angina as the sole drug, or combined with other treatments.

## Patient Preference

Based on federal published guidelines, pharmacological management of chronic angina includes using short acting nitrates for treating the episode of angina, and then combined treatment with long acting nitrates, β-blockers, and Ca^2+^ channel blockers.^57^ As an alternative, ranolazine can also be used by itself or in combination with other anti-anginal drugs. Each one of these drugs has its share of side effects. Ranolazine, however, is the exception in that it brings about its anti-anginal effects without significant clinical changes in hemodynamics (blood pressure, heart rate). Therefore, it may be well suited for patients with lower blood pressures or heart rates in whom the addition or the gradual increase in the dose of anti-anginal drugs with important hemodynamic effects may not be tolerated.^9^ Moreover, ranolazine was shown to have anti-arrhythmic^58^ and anti-diabetic effects.^59^ The latter is of a particular interest since many patients who suffer from chronic angina already have diabetes mellitus. Ranolazine in fact was shown to reduce HbA_1C_ in patients with diabetes mellitus and to possibly mitigate new hyperglycemia in patients at risk for diabetes mellitus. These findings make ranolazine an attractive anti-anginal treatment for patients with chronic angina and impaired glucose metabolism.^59^

Ranolazine costs considerably more than the other anti-anginal drugs per dose. However, and as discussed earlier, a comprehensive retrospective claims analysis^49^ showed that adding ranolazine as a replacement for another ineffective anti-anginal drug was associated with lower rates of prescription fills for short acting nitrates, reduced revascularization rates, and lower total cost of care greater than with other anti-anginal therapies (discussed above, see section Costs and Clinical Outcome). Therefore, the associated reductions in non-pharmacy costs of care when using ranolazine more than offset the higher prescription drug costs.^49^

## Place in Therapy

The FDA approved the use of ranolazine as a first line drug for the treatment of chronic angina. However, the European Society of Cardiology (ESC) still recommends using ranolazine as an add-on therapy or as a substitution therapy when conventional drugs are not tolerated.^2^ Recent guidelines from the American College of Cardiology Foundation and the American Heart Association (ACCF/AHA, etc) also recommends using ranolazine in circumstances in which other anti-anginal drugs are not adequately effective or are not tolerated.^60^ Specifically, ranolazine can be used to relieve symptoms in patients with stable ischemic heart disease in two cases: (1) as a substitute for β-blockers if initial treatment with β-blockers leads to unacceptable side effects or is ineffective or if initial treatment with β-blockers is contraindicated; (2) in combination with β-blockers if the initial treatment with β-blockers is not successful. Ranolazine is available as extended-release tablets at 500 mg and 1000 mg, and a recommendation is to start ranolazine at 500 mg twice daily and increase to 1000 mg twice daily, as needed, based on clinical symptoms. The MERLIN-TIMI 36 trial showed no benefit of ranolazine in treating acute coronary syndrome; therefore ranolazine is not approved for treating these conditions. Clinical trials did not show significant differences in the efficacy of ranolazine between younger and older patients. However, older patients may have a greater frequency of decreased hepatic or renal function and therefore it is recommended to start ranolazine at the lowest dosing range in this population. Ranolazine should not be used in patients with cirrhosis, severe renal insufficiency, preexisting long QT_c_, or in patients using drugs that are known to prolong QT_c_ or inhibit CYP3A4.

## Conclusions

Coronary heart disease remains a global malady despite the advancement in its management. Chronic angina is one manifestation of CAD. Traditional anti-anginal therapy includes drugs that decrease O_2_ demand and increase blood flow. Ranolazine, however, belongs to a different class of anti-anginal drugs. It antagonizes the disturbance in ion homeostasis, specifically Na^+^ and subsequently Ca^2+^ that may occur during ischemia by inhibiting the *I*Na_L_. Other possible mechanisms including its direct effect on cardiac myofilaments, or indirectly preserving mitochondrial complex I, may in part mediate its effects as an anti-anginal drug. Although ranolazine is more expensive than other traditional treatments per dose, it actually cuts the non-pharmacy costs of care in the long run. However, it remains important to compare total costs of care of ranolazine with the other anti-anginal drugs when ranolazine is used as a new, first line, anti-anginal drug and not only as an alternative therapy when the other treatments fail to control angina. Clinical trials showed ranolazine to lack many of the hemodynamic side effects observed with the other anti-anginal drugs, and more importantly to be an effective anti-anginal treatment when used alone or in combination with other conventional treatments. Yet, a direct comparison between ranolazine and the other conventional anti-anginal drugs (β-blockers, Ca^2+^ channel blockers, nitrates) is warranted before ranolazine can seriously be recommended as a first line therapy for chronic angina.

## Figures and Tables

**Table 1:** A summary of the possible mechanisms of ranolazine as an anti-anginal drug.

Mechanism	Details	Notes
Effects on metabolism	Ranolazine increases the amount of active dephosphorylated pyruvate dehydrogenase Ranolazine stimulates glucose oxidation at the expense of fatty acid oxidation	Concentration >10 μM (higher than used in clinical settings) This mechanism suggests an intra-mitochondrial effect
Effects on late Na^+^ current (*I*Na_L_)	Ranolazine inhibits *I*Na_L_ and therefore reduces Na^+^ overload during ischemia This reduces cytosolic Ca^2+^ overload that occurs by activation of Na^+^/Ca^2+^ exchanger in the reverse mode This also reduces mitochondrial Ca^2+^ overload	Ranolazine inhibits *I*Na_L_ with an IC_50_ = 5.9 μM (within the clinical range) Ranolazine inhibits *I*Na_L_ possibly by inhibiting the mechano-sensitivity of Na*v*1.5
Effects on mitochondrial complex I	Ranolazine inhibits mitochondrial complex I Ranolazine reduces electron flow to complex III during ischemia Ranolazine reduces mitochondrial free radical generation	Ranolazine is a weak inhibitor of complex I in coupled mitochondria Ranolazine becomes more potent as a complex I blocker in uncoupled or broken mitochondria
Effects on myofilaments Ca^2+^ sensitivity	Ranolazine modulates myofilament cross-bridge kinetics and sensitivity to Ca^2+^ Ranolazine improves diastolic function at the cardiomyocyte level	The effects on myofilaments are achieved with concentrations of ranolazine within the clinical range

**Table 2:** A summary of the differences between ranolazine and the other traditional anti-anginal drugs in total costs of care and rate of revascularization in the pre- and post-index period.

	Ranolazine (n = 881)	Nitrates (n = 1788)	β blockers/Ca^2+^ channel blockers (n = 1876)
pre-index total cost $	19199	19841 (*P* = 0.603 vs. ranolazine)	18152 (*P* = 0.410 vs. ranolazine)
% pre-index revascularization	24.97	26.51 (*P* = 0.394 vs. ranolazine)	25.16 (*P* = 0.915 vs. ranolazine)
Unadjusted post-index total cost $	14781	17773 (*P* = 0.022 vs. ranolazine)	16785 (*P* = 0.120 vs. ranolazine)
% unadjusted post-index revascularization	9.88	20.25 (*P* < 0.001 vs. ranolazine)	15.51 (*P* < 0.001 vs. ranolazine)
adjusted post-index total cost $	13961	18166 (*P* < 0.001 vs. ranolazine)	17612 (*P* = 0.002 vs. ranolazine)
% adjusted post-index revascularization	9.9	20.4 (*P* < 0.001 vs. ranolazine)	15.4 (*P* < 0.001 vs. ranolazine)

Table assembled from Phelps et al. Clinical Therapeutics/volume 34, Number 6, 2012.
